# Optimization of FDM Printing Parameters for Enhanced Compressive Performance of 3D-Printed PLA/CF Composite Lattice Structures

**DOI:** 10.3390/polym18141696

**Published:** 2026-07-09

**Authors:** Mustafa Saleh, Saqib Anwar, Abdulrahman M. Al-Ahmari, Abdelaty E. Abdelgawad, Najeeb Al-khalli, Abdullah Yahia AlFaify

**Affiliations:** 1 Industrial Engineering Department, College of Engineering, King Saud University, Riyadh 12372, Saudi Arabia; 2 Electrical Engineering Department, College of Engineering, King Saud University, Riyadh 12372, Saudi Arabia

**Keywords:** additive manufacturing, FDM printing parameters, polymer composite, carbon fiber-reinforced PLA, TPMS Lattice Structures, compression testing, specific energy absorption, RSM, optimization

## Abstract

This study statistically examines how fused deposition modeling (FDM) parameters influence the mechanical behavior of FDM-printed lattice structures. Diamond triply periodic minimal surface (D-TPMS) lattice structures were 3D-printed using carbon fiber-reinforced polylactic acid (PLA/CFs) composites. The effects of FDM parameters, including extruder temperature (ET), printing speed (PS), and layer thickness (LT), on the mechanical behavior of D-TPMS structures were investigated using response surface methodology (RSM). Uniaxial compression testing was performed to evaluate the mechanical properties of the 3D-printed samples, including compressive modulus (E), peak strength (σ_peak_), and specific energy absorption (SEA). The optimal FDM parameter settings for maximizing E, σ_peak_, and SEA were determined using multi-objective optimization via the desirability function. A deformation analysis was further conducted. The as-built D-TPMS samples generally matched the design relative density (44%), with absolute errors of 0.3–4.5%, while the largest deviation (~4.5% below the design value) occurred at low-ET and high-LT combinations. The results showed that LT was the dominant factor affecting E and σ_peak_, accounting for 77.45% and 89.25% of the total variation, respectively, whereas ET had the most significant influence on SEA, accounting for 55.76% of its total variation. In addition, increasing ET improved interfacial bonding and shifted the failure mode from early wall and layer fracturing to predominantly wall yielding, thereby enhancing structural integrity during compression. Higher LT deteriorated the mechanical properties (E, σ_peak_, and SEA) and promoted a progressive failure mode characterized by gradual interlayer separation. The findings revealed that the optimal settings (60 mm/s PS, 232 °C ET, and 0.2 mm LT) simultaneously maximized E (0.567 GPa), σ_peak_ (15.937 MPa), and SEA (15.510 J/g), with high predictive accuracy (maximum % error ~±1.41%). Correlation analysis further revealed significant relationships between as-built relative density and the compression responses E, σ_peak_ and SEA, with correlation coefficients exceeding 0.8. Overall, this study advances the understanding of how FDM printing parameters govern the mechanical behavior of PLA/CFs D-TPMS lattice structures and highlights the potential for predicting their mechanical performance.

## 1. Introduction

Lattice structures are lightweight, porous structures that have attracted significant attention in various applications owing to their unique combination of geometric, mechanical, and physical properties. Their internal architecture can be adjusted to satisfy customized functional requirements by enabling control over properties such as architecture, mechanical properties, energy absorption, and heat dissipation [1]. The combination of multi-functionality, design flexibility, and material efficiency has promoted the widespread use of lattice structures. The fundamental feature of lattice structures is that material is only placed where it is structurally or functionally required [2], thereby reducing weight while maintaining sufficient mechanical performance [3]. This efficient material utilization results in high strength-to-weight and stiffness-to-weight ratios, contributing to the widespread use of lattice structures in lightweight structural applications, particularly in the aerospace [4] and automotive [5]. Lattice structures with controlled microarchitectures and porosity can promote tissue integration in biomedical applications [6]. Consequently, they are well-suited for bone-substituting biomaterials and implants, as they enable the fabrication of medical implants with the necessary mechanical properties, biocompatibility, and a porous architecture [2,7]. In addition, the interconnected cellular topology of lattice structures enables the efficient distribution of impact loads [8], thereby improving shock absorption and protection [9]. Overall, lattice structures are widely used in aerospace, automotive, protective, and biomedical applications due to their high performance-to-weight ratio, energy-absorption capabilities, and customizable architectures.

Conventional manufacturing processes have generally limited the production of complex geometries, such as lattice structures. Additive manufacturing (AM) processes, which utilize layer-by-layer production, enable the fabrication of such complex geometries. The design freedom enabled by AM, together with the multifunctional capabilities of lattice structures, has facilitated their widespread adoption in advanced engineering applications. Furthermore, key lattice structure factors, including geometry, material composition, and relative density, can be effectively controlled through AM. Consequently, AM creates new opportunities for the design and fabrication of lattice structures. Numerous AM technologies have been developed, each offering distinct capabilities in terms of material compatibility, dimensional accuracy, surface quality, mechanical performance, production speed, operational simplicity, and cost-effectiveness. The International Standard Organization/American Society for Testing and Materials Standard (ISO/ASTM 52900:2021) classify the AM technologies into seven main categories [10]: binder jetting (BJT), direct energy deposition (DED), material extrusion (MEX), material jetting (MJT), powder bed fusion (PBF), sheet lamination (SHL), and vat photopolymerization (VPP). MEX, also known as fused deposition modeling (FDM), remains the most widely used AM process [11]. FDM is a polymer-based AM process in which thermoplastic materials, in the form of filament, are melted and deposited layer by layer to fabricate a 3D part. The filament is fed into an extruder, which heats and melts the filament to a predetermined temperature. The melted material is then deposited on a building bed, and FDM printing continues layer by layer, with each layer bonding to the previous one. Compared with other polymer-based AM technologies, such as VPP and selective laser sintering (SLS), a PBF process, FDM offers advantages including low cost, operational simplicity, material diversity and affordability, low maintenance requirements, and material recyclability. Among these advantages, material versatility is particularly noteworthy, as FDM can process diverse polymer-based materials, including neat polymers [12,13], polymer blends [14,15], and reinforced polymer composites (RPCs) [16,17,18,19]. The incorporation of reinforcements, such as fibers (e.g., carbon fibers (CFs) and glass fibers (GFs)) or particles (e.g., graphene nanoplatelets), into polymers to produce RPCs in FDM can significantly enhance the mechanical properties of printed parts, making them well-suited for structural and load-bearing applications across various industries [11,20]. These advantages make FDM a promising AM technology for the cost-effective fabrication of structural lattices.

Controlling AM process parameters is essential to meet the engineering requirements of lattice structures [21]. The influence of AM processing parameters on lattice structure performance has been reported for different AM processes, such as SLM [21,22], EBM [23], L-PBF [24], and DLP [25]. Few studies have investigated the influence of FDM printing parameters on the mechanical performance of lattice structures. Wambua et al. [26] studied the influence of layer thickness, printing speed, and bed temperature on the mechanical performance of four-point star-shaped lattice structures using a Taguchi L9 design. Their findings showed that the highest yield strength and modulus of the FDM-printed PLA-based structures were obtained at a high layer thickness (0.3 mm), high printing speed (80 mm/s), and moderate bed temperature (60 °C), with layer thickness being the most significant factor. Tang et al. [27] found that the yield strength and plastic platform stress of FDM-printed PLA-based circular structures decreased with increasing printing temperature (200–240 °C) and printing speed (30–60 mm/s), indicating that the best mechanical performance was achieved at a lower printing temperature (200 °C) and lower printing speed (30 mm/s). Rahman et al. [28] investigated the influence of layer thickness (0.1–0.25 mm), printing speed (30–60 mm/s), nozzle temperature (195–210 °C), and bed temperature (50–65 °C) on the mechanical performance of FDM-printed PLA-based cubic lattice structure using a Taguchi L16 design. Their findings showed that the maximum compressive modulus and strength of the structures were obtained at a layer thickness (0.1 mm), printing speed (50 mm/s), nozzle temperature (205 °C), and bed temperature (60 °C). In comparison, the maximum modulus of toughness was obtained at a layer thickness (0.1 mm), printing speed (60 mm/s), nozzle temperature (210 °C), and bed temperature (65 °C). Increasing the layer thickness reduced the compressive modulus, compressive strength, and modulus of toughness, whereas higher printing temperatures enhanced these properties; printing speed had no effect on the compressive modulus but reduced the compressive strength and enhanced the modulus of toughness. A bed temperature of 60 °C was found to be optimal for all responses. Dixit and Jain [29] investigated the influence of layer thickness (0.1–0.30 mm), printing speed (40–60 mm/s), and infill density (50–100%) on the compressive strength of FDM-printed TPU- and PLA-based BCC lattice structures based on the Taguchi design. The findings showed that the highest compressive strength was obtained at a lower layer thickness (0.1 mm), lower printing speed (40 mm/s), and higher infill density (100%). While printing speed showed no statistical significance on the compressive strength of both PLA- and TPU-based structures, its effect was controversial for both materials. For instance, the strength of PLA-based structures decreased with increasing printing speed, whereas for TPU it first decreased and then increased. Similar work by [30] studied the influence of layer thickness (0.1–0.30 mm), printing speed (30–90 mm/s), and infill density (60–100%) on the compressive strength of FDM-printed PLA-based octet lattice structures using a Taguchi design. Infill density was the most significant factor, followed by printing speed and layer thickness. The highest compressive strength per mass was obtained at a layer thickness of 0.2 mm, printing speed of 90 mm/s, and an infill density of 100%. In this study, layer thicknesses of 0.1 mm and 0.2 mm yielded comparable results; however, the compressive strength per mass decreased significantly at 0.3 mm. The compressive behavior of truncated octahedron and cubic diamond lattice structures was statistically evaluated in relation to geometrical parameters (cell type, cell size, strut diameter), material type (ABS and PC), and FDM process parameter (layer thickness) [31]. The results showed that the design parameters had the most significant influence on the plateau stress and elastic modulus of the lattice structures. In contrast, material type and layer thickness had limited effects. Increasing the layer thickness (0.15–0.25 mm) enhanced the plateau stress without affecting the structure’s stiffness.

Lattice structures are advantageous because material is placed only where it is needed. This implies that factors such as material-inherent properties, lattice design, material amount (relative density), and manufacturing process determine the performance of lattice structures. While the influence of lattice geometry and relative density on the lattice’s performance is well documented, the majority of reported studies on lattice structures have overlooked other potentially relevant factors, such as FDM printing parameters. The reported studies on FDM printing parameters focused solely on single polymers with no reports on composite polymers [26,27,28,29,30,31]. Furthermore, the analysis was conducted using either a one-factor-at-a-time approach or a Taguchi design, both of which are limited to identifying main effects and do not provide polynomial predictive models. Previous studies have primarily focused on strut-based lattice structures, while the effects of FDM printing parameters on surface-based lattice structures (e.g., TPMS lattices) have received limited attention. To the best of the authors’ knowledge, there has been no systematic investigation and optimization of the effects of the lattice design parameters, relative density, material composition, and the FDM printing parameters on the mechanical performance of surface-based lattice structures. In our previous works [32,33], we extensively investigated the influence of material compositions (e.g., pure PLA and PLA/CFs), design parameters (different TPMS cell topologies and cell sizes), and various RDs on the mechanical behavior of the surface-based TPMS structures. Also, these parameters were optimized for enhancing the mechanical properties of the FDM-printed surface-based TPMS structures. Therefore, in this current work, the optimal design addressed in [32] (i.e., a diamond lattice structure with 12 mm cell size and 44% RD at 15% CFs) is considered to further investigate the influence of the FDM printing parameters on the mechanical properties of the composite TPMS lattice structures. Accordingly, this study aims to examine the effects of various FDM printing parameters on the mechanical performance of TPMS lattice structures manufactured from a polymer composite. This research statistically examines how FDM parameters, including extruder temperature (ET), printing speed (PS), and layer thickness (LT), influence the mechanical characteristics of FDM-printed TPMS lattice structures. The analysis was conducted based on a response surface methodology (RSM) approach. Uniaxial compression testing was carried out to evaluate the mechanical properties of 3D-printed samples, including compression modulus, strength, and SEA. The best FDM parameter settings for maximizing the TPMS lattices’ performance were determined using multi-objective optimization via the desirability function. Moreover, the failure mechanisms were explored through deformation analysis. The morphology of the FDM-printed TPMS composite structures was also evaluated using a scanning electron microscope (SEM).

## 2. Materials and Methods

### 2.1. Materials

In this study, a carbon-fiber-reinforced PLA (PLA/CFs) composite filament with 1.75 mm diameter was used to 3D-print the lattice structures. The PLA/CFs contains a biopolymer-grade PLA (Natureworks 4043D PLA) with 15% CFs. The PLA/CFs was obtained from 3DXTech (Grand Rapids, MI, USA) and its characteristics are illustrated in Table 1, as reported in the material data sheet [34].

**Figure 1:**
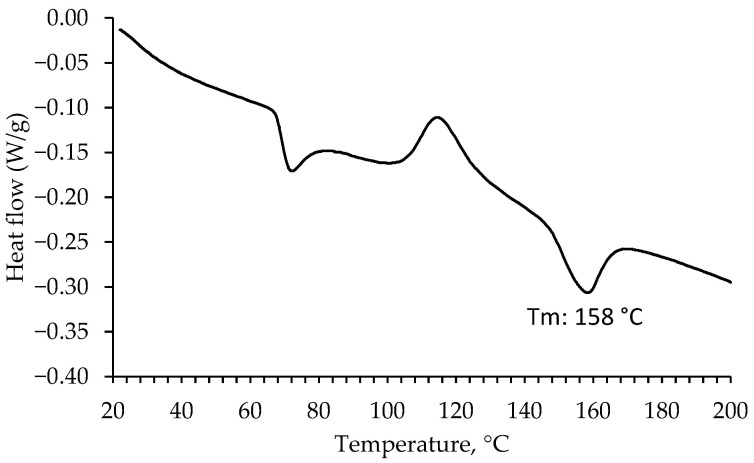
DSC thermal characteristics of the PLA/CFs.

The thermal characteristics of PLA/CFs, including the glass transition temperature (Tg), cold crystallization temperature (Tcc), and melting temperature (Tm), were determined using differential scanning calorimetry (DSC) with an LR-STA-200 (Lonory, Dongguan, China). The DSC analysis was conducted at a heating rate of 10 °C/min from ambient temperature to 200 °C. The sample was placed in an aluminum crucible under a nitrogen (N_2_) atmosphere. According to the DSC curve shown in Figure 1, the Tg, Tcc, and Tm of the PLA/CFs are 69 °C, 115 °C, and 158 °C, respectively.

### 2.2. Lattice Structure

In this study, diamond TPMS (D-TPMS) lattice structures were considered. The D-TPMS structures were designed with dimensions of 24 mm × 24 mm × 48 mm [32,33], adhering to the guidelines of ASTM D695-15 [35], a standard test method for assessing the compressive properties of rigid polymer materials.

**Figure 2:**
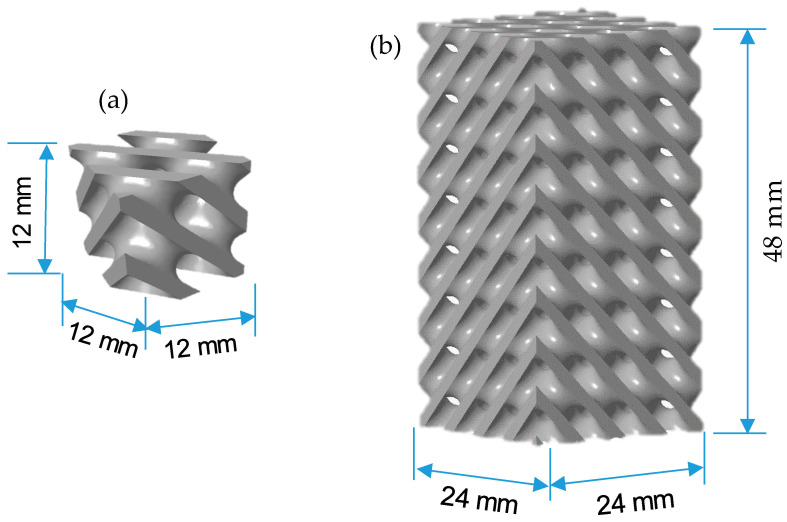
Schematic illustration of the D-TPMS lattice structure: (**a**) a single D-TPMS unit cell with a cell size of 12 mm and (**b**) the corresponding D-TPMS lattice structure.

The optimal TPMS lattice structure identified in [32], which features a diamond TPMS lattice topology with a cubic unit cell with an edge length of 12 mm (Figure 2a), and 44% relative density (RD) at 15% carbon fibers (CFs), is used for this study. The specimens had overall dimensions of 24 mm × 24 mm × 48 mm (Figure 2b), resulting in a 2 × 2 × 4 array of unit cells, i.e., two unit cells along each side of the base and four unit cells along the height. As discussed in our previous work [32], this design was chosen based on findings that demonstrated maximized mechanical performance, including compressive modulus, compressive strength, and specific energy absorption. Consequently, the D-TPMS structures were designed with a 12 mm cell size and 44% RD, and were 3D-printed using PLA/CFs containing 15% CFs.

The needed relative density (RD, i.e., 44%) was determined and measured based on the volume fraction, i.e., the lattice structure’s volume divided by the overall structure’s volume. The RD of a structure is a function of the wall thickness and the cell size (e.g., 12 mm in the considered design). Thus, the D-TPMS wall thickness parameter (t) was set to 1.1786 in the CREO 8.0 software to obtain 44% RD. The D-TPMS structure was designed using CREO 8.0 software. Figure 2 depicts the D-TPMS cell topology and the corresponding D-TPMS structure.

### 2.3. Experimental Design and FDM Printing

This research statistically examines how FDM parameters, including printing speed (PS), extruder temperature (ET), and layer thickness (LT), influence the mechanical behavior of FDM-printed D-TPMS lattice structures. PS is the speed at which the nozzle travels along the *X* and *Y* axes while depositing the melted material. ET refers to the temperature at which the filament is heated in the extruder before the material is deposited on the printing bed. LT is the height of a single layer (along the *Z*-axis) of material deposited by the nozzle.

Table 2 presents the FDM parameters (PS, ET, and LT) along with their corresponding levels. The range of the considered parameters was selected based on preliminary experiments, supplier recommendations, and the FDM printer’s capabilities. For instance, the range of the extruder temperature for the PLA/CFs, as recommended by the supplier, is (190–220 °C). However, based on the preliminary experiments, an extruder temperature below 205 °C causes clogging during printing. Clogging can occur because CF reinforcements increase viscosity and, consequently, reduce flow, particularly at lower temperatures. At lower temperatures, clogging was evidenced by extruder clicking sounds, discontinuous filament deposition with gaps between layers, and, in some cases, complete nozzle blockage without material deposition. Furthermore, to avoid nozzle wear, we used a hardened steel nozzle, which requires a higher extrusion temperature due to its lower thermal conductivity compared to the brass nozzle (commonly used). Similarly, the supplier recommended an LT exceeding 0.20 mm, since smaller LT values may lead to nozzle clogging during PLA/CFs printing. An insufficiently small gap between the nozzle and the previous layer/printing bed may promote CF agglomeration, thereby preventing or limiting the deposition of the molten material. The lattice specimens (24 mm × 24 mm × 48 mm) were fabricated in a vertical-build orientation, with the 48 mm height aligned parallel to the printer’s *Z*-axis. The other FDM process parameters that were kept constant throughout the experiments are listed in Table 3.

**Figure 3:**
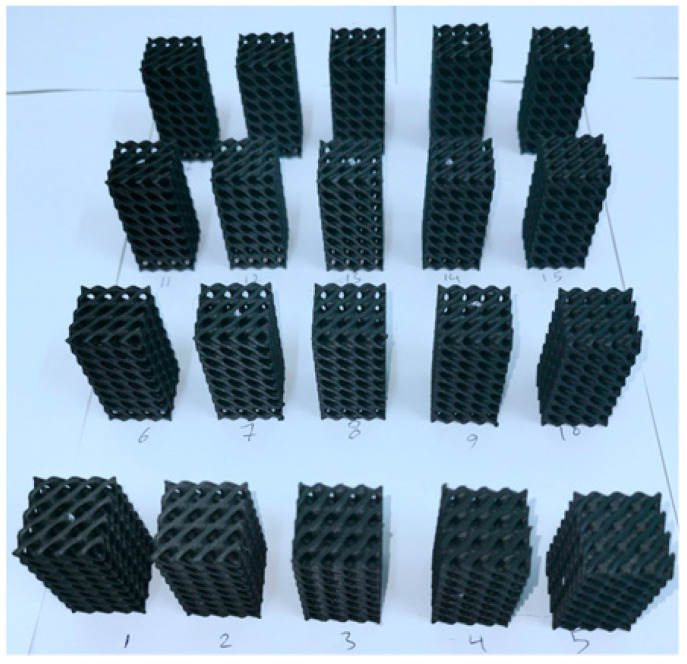
Samples of FDM-printed TPMS structures corresponding to the RSM runs.

The D-TPMS composite structures were 3D-printed using a Prusa FDM printer (Prusa i3 MK3S+, Prague, Czech Republic) fitted with a 0.4 mm hardened steel nozzle. PrusaSlicer 2.4.2 was used to generate the GCODE files for the 19 RSM runs. Samples were randomly FDM-printed. Examples of the printed D-TPMS lattice structures are shown in Figure 3.

Uniaxial compression testing was carried out to evaluate the mechanical properties of the 3D-printed samples, including compression modulus (E), compressive peak strength (σ_peak_), and specific energy absorption (SEA), as described in Section 2.5. The statistical analysis was conducted using a design-of-experiments approach, namely response surface methodology (RSM). Based on the RSM design, 19 experiments were conducted and repeated twice, resulting in a total of 38 experiments. ANOVA was performed at the 95% confidence level to assess the influence of the FDM variables and their interactions on E, σ_peak_, and SEA. *p*-Values of less than 0.05 impsly that model terms (main factors and interactions) are statistically significant. Prediction models for the responses were also developed and employed to optimize the FDM parameters. Reduced models were developed by eliminating statistically non-significant terms using elimination-based selection algorithms, thereby improving prediction accuracy without sacrificing model fit. The best FDM parameter settings that simultaneously maximize the D-TPMS lattices’ mechanical properties (E, σ_peak_, SEA,) were determined using multi-objective optimization via the desirability function. Statistical analysis was performed utilizing Design-Expert 13 software.

### 2.4. Metrological Characterization

The dimensions of the fabricated samples, including width, thickness, and length, were assessed using a Multi-Lens Vertical Lab Profile Projector (model VOM-2515, Guangdong Micro Accuracy Co., Ltd., Dongguan, China). The actual (as-built) RD of the samples was determined using Archimedes’ principle. For the density measurements, a Shimadzu Analytical Balance (AUW220D, Kyoto, Japan) with a precision of 0.01 mg was utilized, alongside a universal specific gravity kit (SGK-C, Mineralab, Bedford, UK). For RD measurements, a single-cell of D-TPMS lattice with 44% RD and 12 mm size was designed and FDM-printed based on the 19 RSM runs.

**Figure 4:**
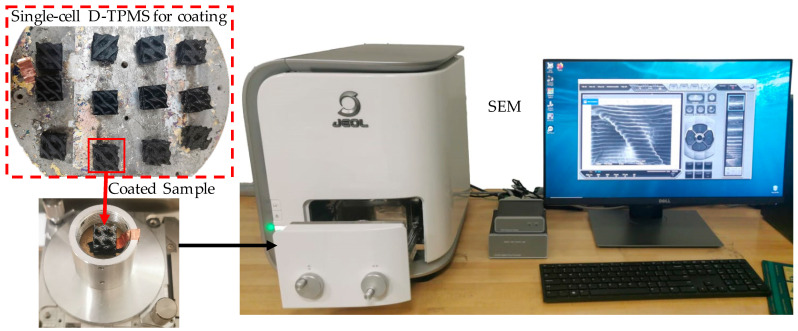
Morphological characterization of the D-TPMS samples using SEM.

The morphology of the FDM-printed D-TPMS structures was evaluated via a scanning electron microscope (SEM) from JEOL (JCM 6000 plus, Tokyo, Japan). Single-cell D-TPMS structures coated with aluminum were used for morphological analysis, as shown in Figure 4.

### 2.5. Mechanical Properties

**Figure 5:**
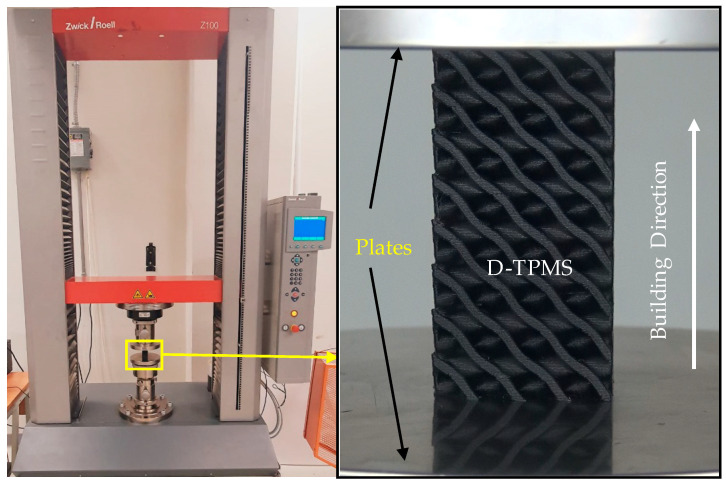
Compression testing setup.

Uniaxial compression testing was conducted in accordance with the ASTM D695-15 standard [35]. Compression tests were performed on the 3D-printed D-TPMS lattice samples at a constant crosshead speed of 1.6 mm/min until a strain of 60% was reached. A Zwick Z100 universal testing machine, equipped with a 100 kN load cell, was utilized, as illustrated in Figure 5. Compression characteristics, including E, σ_peak_, and SEA, were gathered based on the stress–strain curves. σ_peak_ was considered as the first maximum peak strength in the stress–strain curve. A lattice structure’s SEA was determined by dividing the area under the force–displacement curve up to 55% nominal strain, i.e., theoretical densification strain, by the structure’s weight. Deformation evolution during compression testing was captured via video recording using a digital camera (Sony HDR-PJ820, Tokyo, Japan).

## 3. Results

Table 4 shows experimental results of the E, σ_peak_, and SEA of the 19 RSM runs, along with the variation among the repeated samples, as indicated by the standard deviation (SD).

### 3.1. RD Characteristics

**Figure 6:**
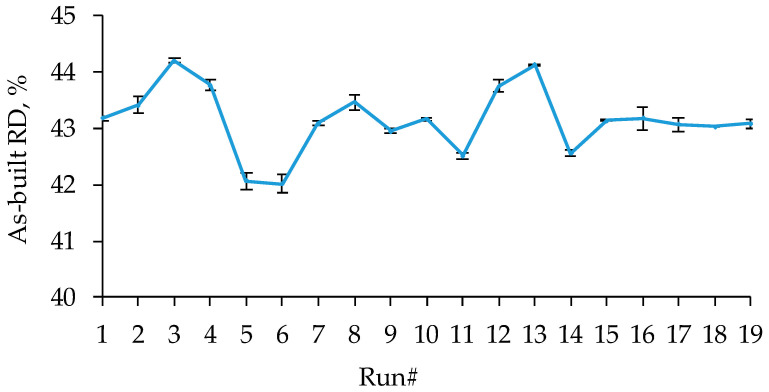
As-built RD of the FDM-printed D-TPMS structures for the 19 RSM runs.

Figure 6 shows the actual (as-built) relative densities of the D-TPMS lattice structures under the considered FDM conditions. The relative densities of the as-built samples were in close agreement with the design values (44%), with a maximum error of ~−4.5%. The largest deviation occurred due to the combination of a low ET (205 °C) and high LT (0.3 mm) in runs #5 and #6. Lowering the ET reduces the composite’s flowability, resulting in less material being deposited. A higher LT increases the gaps between adjacent rasters and layers, resulting in a lower RD. The minimum deviation from the designed RD (i.e., 44%) was found at run#13, with ~0.3% percentage error, followed by runs #3, 4, and 12 with an absolute percentage error of ~0.5%. Run#13 exhibited the minimum deviation from the designed RD (44%), with an error of ~0.3%, followed by runs #3, #4, and #12, which showed an absolute percentage error of ~0.5%.

### 3.2. Mechanical Characterization

**Figure 7:**
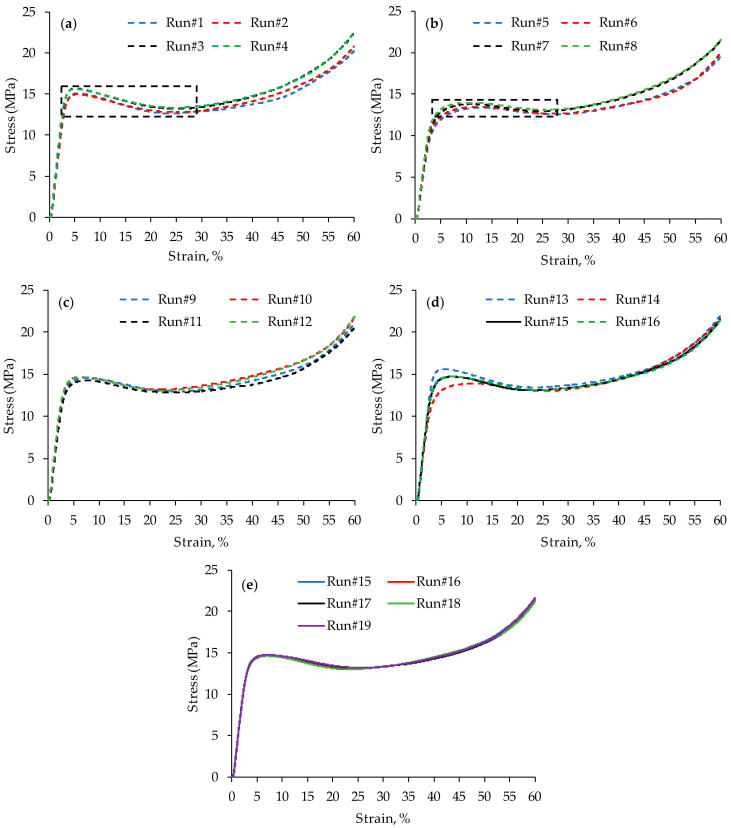
Typical stress–strain curves of the experimental runs listed in Table 4, classified based on the experiment number: (**a**) run#1–run#4, (**b**) run#5–run#8, (**c**) run#9–run#12, (**d**) run#13–run#16, and (**e**) stress–strain curves of the center-point replicates (run#15–run#19). Dotted lines indicate the stress transition before and after the peak strength.

The stress–strain curves under the uniaxial compression test of the 19 runs listed in Table 4 are depicted in Figure 7a–e. Figure 7 shows three stages in the stress–strain curves: (1) the linear elastic compression stage, where the stress–strain curve is linear; (2) the plastic stage, referred to as the plateau stage, which features plastic deformation, wall yielding and fracturing, layer separation, and cell collapse; and (3) the densification stage, where the collapsed walls and cells are compacted, resulting in a rapid increase in stress. The stress–strain curves showed uniform deformation, as evidenced by the absence of significant rises and falls in the stress–strain curves during cell collapse, i.e., during the plateau stage. It is evident that the mechanical behavior of the lattice structures is influenced by the considered FDM parameters, e.g., PS, ET, and LT. This influence is shown by how much the stress–strain curves vary in terms of either the stress range or the shape of the curves. The influence of the LT on the stress range is clearly shown in Figure 7a,b at 0.2 mm and 0.3 mm, respectively, where the load-bearing capacity of structures at 0.2 mm LT is higher. Despite the higher stiffness and peak strength of runs #1–4, printed with a layer thickness (LT) of 0.2 mm, shown in Figure 7a, compared with their corresponding runs #5–8, printed with an LT of 0.3 mm, shown in Figure 7b, runs #5–8 exhibited greater plastic deformation before stress reduction. In contrast, runs #1–4 showed a sharp stress reduction immediately after the elastic deformation, as highlighted by the black rectangles in Figure 7a,b. In other words, the transition before and after the peak strength is less abrupt at the higher LT (0.3 mm) in runs #5–8 (Figure 7b) than in runs #1–4 (Figure 7a). Figure 7a clearly shows the influence of changing ET from 205 °C (run#2) to 235 °C (run #4) at a fixed LT and PS, showing an improvement in the mechanical behavior of the TPMS composite structures throughout the three stages described above. Figure 7c,d illustrate the stress–strain curves of the axial and center-point RSM runs. Figure 7e shows the stress–strain curves of experiments 15–19. It should be noted that these experiments are replicating the center point at the same conditions (i.e., PS: 45 mm/s; ET: 220 °C, and LT: 0.25 mm). The closeness of these experiments (run#15–run#19), as seen in Figure 7e, shows fewer variations among the replicates.

**Figure 8:**
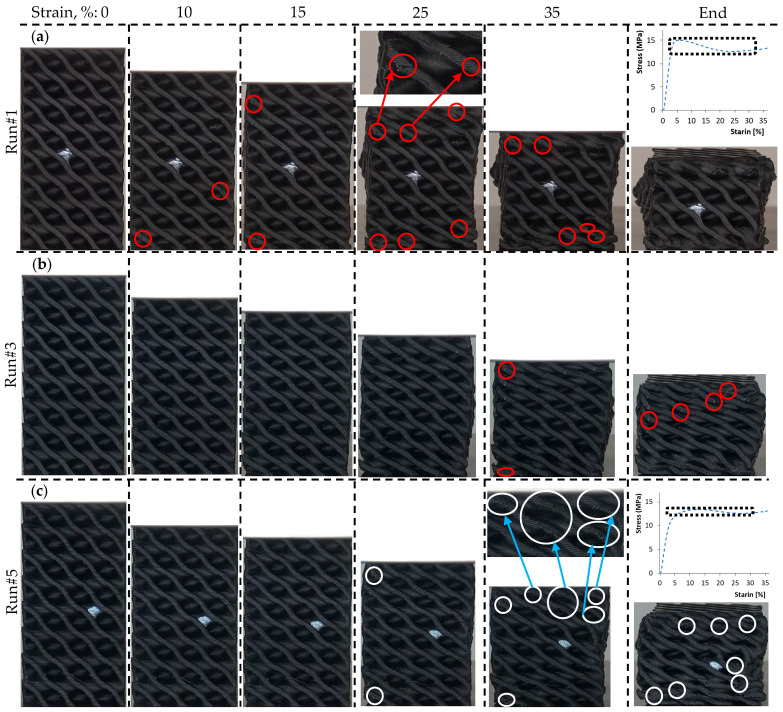
Failure evaluation under compression testing of lattice structures corresponding to: (**a**) Run#1 (30 mm/s PS, 205 °C ET, and 0.2 mm LT), (**b**) Run#3 (30 mm/s PS, 235 °C ET, and 0.2 mm LT), and (**c**) Run#5 (30 mm/s PS, 205 °C ET, and 0.3 mm LT). Red circles indicate cracks and layer fracture; white circles indicate layer separation; dotted lines indicate the stress transition before and after the peak strength.

Figure 8 illustrates the deformation behavior and failure mechanisms of typical lattice structures (Run#1, Run#3, and Run#5) under compression testing. The deformations highlight the effects of ET (Figure 8a,b) and LT (Figure 8a,c) on structural behavior while maintaining PS at a constant 30 mm/s. It is evident that cracks and fracturing initiate much earlier, at around 5% strain, under a low ET (Figure 8a) compared to a high ET, where they begin at around 35% strain (Figure 8b). At a high ET (235 °C) (Figure 8b), fractures in walls and layers are minimal, indicating that deformation occurs primarily through wall yielding rather than fracturing. In contrast, at a lower ET (205 °C), deformation is dominated by walls/layers fracturing, with concentrated failures clearly visible in Figure 8a. These findings highlight how ET influences the failure mechanisms, ranging from D-TPMS wall fracturing at a low ET to wall yielding at a high ET. Lowering ET reduces the composite’s flowability, leading to less material deposition and the formation of micro-gaps or voids that promote crack initiation and propagation, as will be discussed in Section 4.

Comparing 0.2 mm (Figure 8a) and 0.3 mm (Figure 8c) LT, fractures initiate later at the higher LT, at around 25% strain. At a higher LT (0.3 mm), as shown in Figure 8c, the failure pattern appears different: many layers seem to gradually separate, as highlighted by the white circles, rather than undergoing concentrated fracturing at specific layers. This behavior can also be linked to the stress–strain curves shown in Figure 7b, where the region before and after the peak strength does not change sharply, as also highlighted by the black dotted rectangles on the stress–strain graphs in the last column of Figure 8a,c. This confirms the absence of abrupt wall or layer fractures and implies that layer-to-layer bonding gradually weakens, leading to layer separation rather than layer fracturing. This behavior could be attributed to the following reasons: (1) the reduced contact area between rasters due to the increased circularity at high LT (0.3 mm), and (2) a larger raster diameter implies a higher capacity to withstand loads, such as bending and buckling, as well as fracture, thereby promoting gradual layer deformation due to weak bonding rather than localized or sudden fracture.

### 3.3. ANOVA Analysis

**Figure 9:**
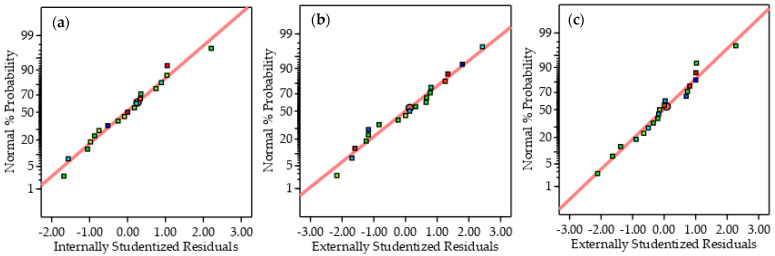
Residual normality plots of: (**a**) E, (**b**) σ_peak_, and (**c**) SEA.

Figure 9 shows the normality plot of the considered responses, showing that the normality assumption is satisfied. The *R*^2^ (as illustrated in Table 5) of E, σ_peak_, and SEA is 99.46%, 99.83%, and 96.02%, respectively, which indicates an excellent representation of the variability of the data by the model terms. Furthermore, the differences between the adjusted and predicted *R*^2^ values are small (see Table 5) and below the commonly accepted threshold of 0.20 (20 percentage points), indicating that the developed models possess good predictive capability and are not overfitted. In addition, Table 5 shows that the adequate precision values for all responses are substantially higher than the recommended minimum value of 4, confirming an adequate signal-to-noise ratio and the suitability of the models for navigating the design space.

#### 3.3.1. Analysis of Compression Modulus

Table 6 shows the reduced ANOVA table of the compressive modulus, E. The ANOVA table shows that all considered factors, including PS, ET, and LT, significantly influence E. The contribution of each significant term is also illustrated in Table 6. The most significant effects are caused by changing the LT variable (76.19% from LT and 1.26% from LT^2^), followed by ET (13.80% from ET and 3.48% from ET^2^), and then the PS (3.08%). The effect of the two-source interactions is minimal, as their contribution to the E variability is low.

#### 3.3.2. Analysis of Compression Strength

The reduced ANOVA table of the peak strength, σ_peak_, is presented in Table 7. The ANOVA table indicates that all studied factors, including PS, ET, and LT, significantly affect E. It is also evident from Table 7 that LT is the most significant factor among the others, as it contributes to 89.02% from LT and 0.23% from LT^2^ of the total variability in σ_peak_, followed by ET (8.88% from ET and 0.6% from ET^2^). Although the PS-LT and ET-LT interactions are significant, their contribution to the variation in σ_peak_ is minimal. For instance, the PS–LT and ET–LT interactions accounted for only 0.24% and 0.35% of the total variation, respectively.

#### 3.3.3. Analysis of SEA

As illustrated in Table 8, the FDM printing parameters (PS, ET, and LT) significantly influence SEA. The ET variable has the largest contribution impact on SEA (48.92% from ET and 6.84% from ET^2^), then the LT (28.89%), and finally the PS (11.25% from PS and 0.12% from PS^2^).

**Figure 10:**
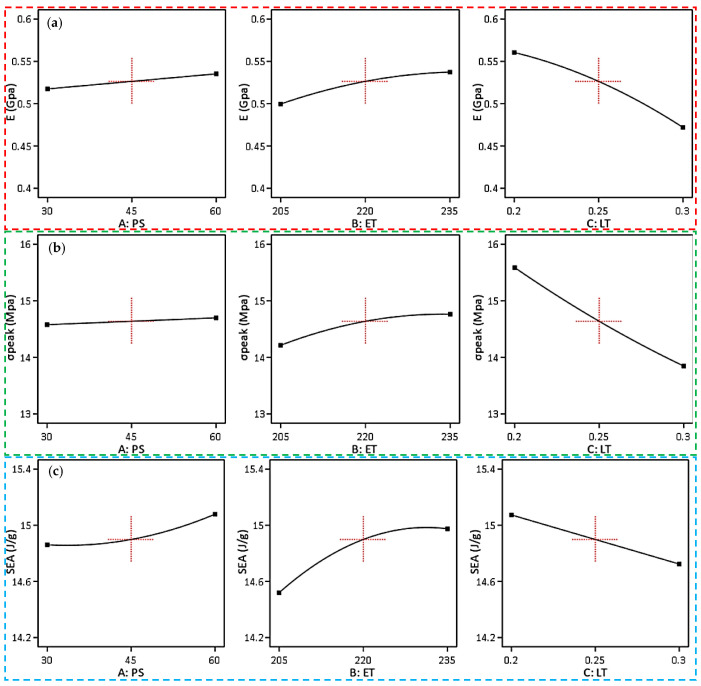
Main effect plots of FDM parameters (PS, ET, and LT) on (**a**) E, (**b**) σ_peak_, and (**c**) SEA.

**Figure 11:**
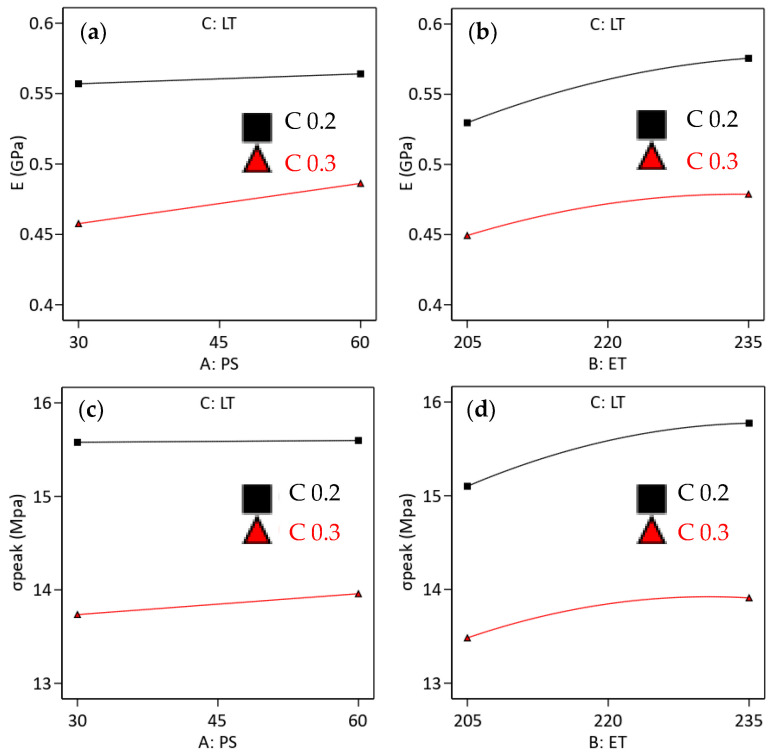
Interaction effects of process parameters on E (**a**,**b**) and on σ_peak_ (**c**,**d**): (**a**) PS-LT interaction, (**b**) ET-LT interaction, (**c**) PS-LT interaction, and (**d**) ET-LT interaction.

Figure 10 provides a visual representation of how the main FDM printing factors impact the E, σ_peak_, and SEA. In general, the directional influence of the FDM printing factors on E (Figure 10a), σ_peak_ (Figure 10b), and SEA (Figure 10c) appears to exhibit a similar pattern. For instance, PS and ET exhibit a proportional influence on E, σ_peak_, and SEA, indicating that a change from a low level to a high level of any of them (PS and ET) leads to an increase in E, σ_peak_, and SEA. On the contrary, LT shows an inverse proportionate effect, whereby an increase in LT results in a decrease in E, σ_peak_, and SEA. Figure 11a,b illustrate the influence of the PS–LT and ET–LT interactions, respectively, on E. Figure 11a indicates a slight interaction between PS and LT on E. Increasing PS leads to a marginal increase in E at both LT levels, although the effect was more pronounced at the LT of 0.3 mm. Similarly, Figure 11c,d illustrate the influence of the PS–LT and ET–LT interactions, respectively, on σ_peak_. Despite their statistical significance, the directional effects of these interactions are not sufficiently clear because their contributions to the total variation in σ_peak_ are very low (PS-LT: 0.24%; ET-LT: 0.35%).

### 3.4. Prediction Modeling

Mathematical relationships between the responses, including E, σ_peak_, and SEA, and the investigated variables (PS, ET, and LT) were developed based on the reduced models in the ANOVA analysis. The developed mathematical models are presented in Table 9.

**Figure 12:**
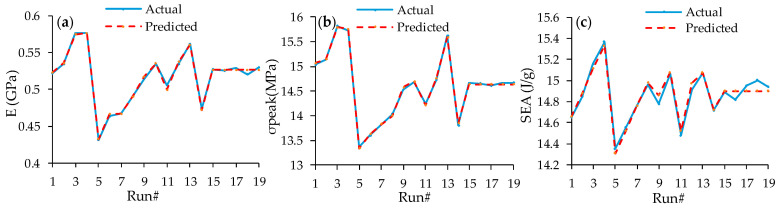
Comparison between the actual (experimental) and the corresponding predicted results of the 19 RSM runs of (**a**) E, (**b**) σ_peak_, and (**c**) SEA.

Figure 12 illustrates a comparison between the experimental (actual) and predicted results for E, σ_peak_, and SEA across the 19 RSM experiments. As can be seen from Figure 12a,b, there is a high degree of agreement between the actual and predicted values for E and σ_peak_, respectively. Similarly, the actual and predicted results of SEA shown in Figure 12c are comparable, with a maximum difference of ~0.1 J/g between the actual and predicted results.

### 3.5. Multi-Objective Optimization

Desirability analysis was used to select the best ET, PS, and LT settings that led to maximizing the E, σ_peak_, and SEA. The following settings should be used to maximize E, σ_peak_, and SEA: the ET should be set at 232 °C, the PS should be set at 60 mm/s, and the LT should be set at 0.2 mm. The optimal structures were FDM-printed, repeated twice, and the average results of E, σ_peak_, and SEA are reported. Table 10 shows the considered variables’ optimal combination values and the experimental validation for multi-objective optimization. The experimentally validated results are consistent with the predicted ones, with an approximate percentage error of ±1% in all responses, as demonstrated in Table 10.

## 4. Discussion

From the results presented in Table 4, lattice structures printed at a high ET and low LT (run#3 and run#4) showed the best mechanical properties, including E, σ_peak_, and SEA. For instance, the maximum compressive modulus (0.577 GPa) and σ_peak_ (15.816 MPa) were observed at 30 mm/s PS, 235 °C ET, and 0.2 mm LT (run#3). Similar findings were observed in (run#4), where the SEA was the maximum (15.366 J/g). On the other hand, the worst mechanical responses were obtained at a low ET, low PS, and high LT (run#5). For example, the minimum E (0.432 GPa), σ_peak_ (13.373 MPa), and SEA (14.351 J/g) were obtained at a low ET, low PS, and high LT (run#5).

**Figure 13:**
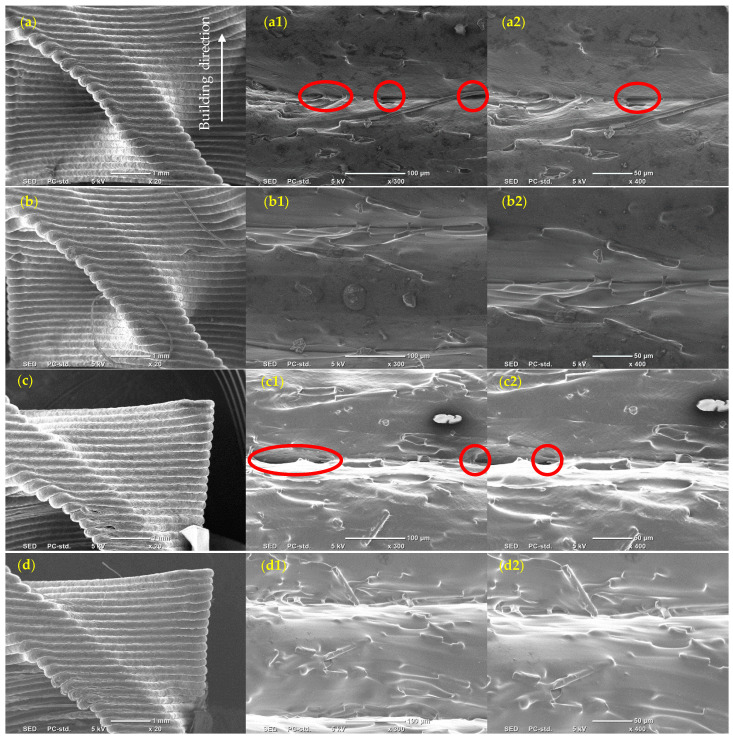
SEM images showing interlayer morphologies of the D-TPMS structures: (**a**–**a2**,**c**–**c2**) Run#1 (30 mm/s PS, 205 °C ET, and 0.2 mm LT) and (**b**–**b2**,**d**–**d2**) Run#3 (30 mm/s PS, 235 °C ET, and 0.2 mm LT). Red circles indicate voids.

**Figure 14:**
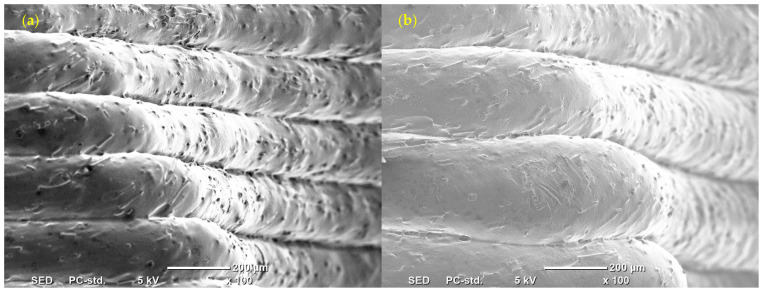
SEM images showing the layers morphologies of D-TPMS structures at different LTs: (**a**) 0.2 mm LT (Run#1: 30 mm/s PS, 205 °C ET, and 0.2 mm LT) and (**b**) 0.3 mm (Run#5: 30 mm/s PS, 205 °C ET, and 0.3 mm LT).

The effect of ET on E, σ_peak_, and SEA was proportional, with an increase in ET from 205 °C to 235 °C enhancing the mechanical performance of the D-TPMS structures. These results are consistent with those reported in [28], where increasing ET from 195 °C to 210 °C improved the compressive modulus and strength of PLA-based structures. However, the findings reported in [27] showed the opposite trend, with the yield strength and plateau stress of FDM-printed PLA-based circular structures decreasing as the printing temperature increased from 200 °C to 240 °C. At low ET (i.e., 205 °C), the composite has high viscosity (low melt flow rate), making it difficult to extrude, as also highlighted by [36]. On the other hand, a higher ET facilitates the flow, which in turn enables the gaps/voids between the adjacent rasters to be filled and reduces the likelihood of clogging. Furthermore, the voids and gaps commonly observed in FDM-printed composite materials can be fully or partially filled due to the overflow of molten material at elevated temperatures. Consequently, the mechanical properties are enhanced due to the filling of the micro-gaps and voids and improve the bonding between intralayers and interlayers. Furthermore, this will improve the SEA by reducing the initiation and propagation of failures, i.e., those caused by voids and insufficient bonding, during the plastic deformation phase, i.e., the region in the stress–strain curve that accounts for the majority of the SEA. This behavior is illustrated in Figure 8, where structures fabricated at a lower extrusion temperature exhibit earlier crack initiation and fracture at approximately 5% strain (Figure 8a), whereas specimens fabricated at a higher extrusion temperature maintain structural integrity until approximately 35% strain (Figure 8b). Because SEA depends on the material’s ability to sustain deformation and absorb energy over a large strain range (up to 55% strain in the present study), the enhanced bonding associated with higher extrusion temperatures has a more pronounced effect on SEA than on E or σ_peak_. This explains why extrusion temperature exhibits the highest contribution to SEA, whereas layer thickness is more influential for E and σ_peak_. For instance, ET accounted for 55.76% (48.92% from ET and 6.84% from ET^2^) of the variability in SEA, whereas LT accounted for 28.89%. Figure 13 compares the interlayer morphologies of structures printed at a low ET (Run#1; Figure 13a,c) and high ET (Run#3; Figure 13b,d), considering different locations. A dominant presence of deep voids and gaps is observed at the low ET (205 °C) compared with the high ET (235 °C), as highlighted by the circles. It should be highlighted that a further increase in the ET may degrade the mechanical behavior, as evidenced from the main effect plots in Figure 10, particularly the peak strength (Figure 10b) and SEA (Figure 10c). This could be attributed to the fact that increasing ET further causes viscosity to substantially decrease, which in turn lowers the structure’s strength [37]. Regarding LT, the ANOVA results showed that LT had the greatest influence on both E and σ_peak_, contributing 77.45% of the total variation in E (76.19% from LT and 1.26% from LT^2^) and 89.25% of the total variation in σ_peak_ (89.02% from LT and 0.23% from LT^2^), respectively. Increasing LT from 0.2 mm to 0.3 mm decreased the mechanical performance of the D-TPMS composite structures. These findings are consistent with those reported in [28], which also showed that LT had the highest influence on the compressive modulus and strength, where a lower LT resulted in improvements in these properties. While the findings reported in [26] also identified LT as the most significant factor, they showed the opposite trend, as the highest yield strength and modulus of the FDM-printed PLA-based structures were achieved at a higher LT (0.3 mm). A high LT (e.g., 0.3 mm) results in the deposition of rasters with a more rounded shape (Figure 14b) compared to the less rounded rasters observed at a low LT (Figure 14a). Consequently, more gaps with a reduced contact area between adjacent rasters and layers are formed at a high LT. This, in turn, leads to the initiation and propagation of cracks during compression testing. In contrast, a low LT (e.g., 0.2 mm) creates denser parts and better layer-to-layer bonding, leading to better mechanical properties. PS showed a relatively lower impact on E and SEA, contributing 3.08% and 11.37% (11.25% from PS and 0.12% from PS^2^) of the total variation, respectively, while its effect on σ_peak_ was marginal. In general, increasing PS from 30 mm/s to 60 mm/s enhanced the mechanical performance of the D-TPMS composite structures. The literature reports conflicting findings regarding the effect of PS on the mechanical behavior of lattice structures. For instance, for PLA-based lattice structures, Ref. [26] reported an increase in the compressive modulus at a higher PS (80 mm/s), while [30] observed an improvement in the compressive strength at a higher PS (90 mm/s). In contrast, Ref. [27] reported a decrease in the yield strength and plastic platform stress as PS increased from 30 mm/s to 60 mm/s. Another study, Ref. [28], showed that PS had no effect on the compressive modulus but reduced the compressive strength.

**Figure 15:**
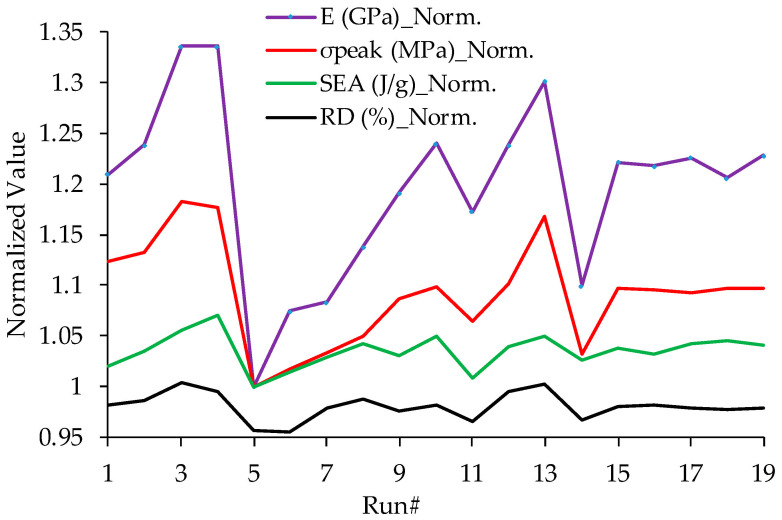
A comparison between the mechanical responses (E, σ_peak_, and SEA) and as-built RD.

As previously mentioned, the high ET promotes the flow, while the lower LT reduces the roundness of the raster. This directly contributes to the production of dense structures with minimal voids/gaps, as assessed by the as-built RD. For instance, at 235 °C ET and 0.20 mm LT (run#3), the as-built RD of the D-TPMS structure is 44.17%, while at 205 °C ET and 0.30 mm LT (run#5), the as-built RD is 42.06%. Figure 6 and Figure 15 illustrate the as-built RD of the FDM-printed D-TPMS structures, highlighting the as-built RD variations among the RSM runs listed in Table 4. It is important to note that the as-built RD shown in Figure 15 was represented in “normalized values” by dividing the as-built RD by the intended one, 44%. As evident from Figure 6 and Figure 15, the minimum as-built RDs were observed for runs #5, 6, 11, and 14, where the ET was low and/or the LT was high, indicating that both parameters (ET and LT) significantly impact the as-built RD. The RD is the most significant factor influencing the mechanical behavior of lattice structures, and any deviation from the designed RD will certainly affect the mechanical behavior, as also illustrated by [32,33]. Figure 15 also presents normalized values of E, σ_peak_, and SEA to further illustrate the correlation between them and the as-built RD. Please note that the normalized values for each response of E, σ_peak_, and SEA were calculated by dividing the corresponding result by its corresponding minimum value. It is evident from Figure 15 that the as-built RD is correlated with all responses, where a higher as-built RD indicates improved mechanical performance and vice versa. The Pearson correlation test confirms the relationship between RD and all responses, as illustrated in Table 11, demonstrating a significant correlation with a high coefficient (higher than 0.8). Although all specimens were designed with an identical relative density (44%), the as-built RD varied as a function of the considered FDM-printing parameters, particularly ET and LT. High correlations were observed between RD and the mechanical responses (E: r = 0.839; σ_peak_: r = 0.854; SEA: r = 0.818), indicating that RD variations contributed substantially to the mechanical behavior. Nevertheless, the FDM printing parameters may also influence the mechanical response through RD-independent mechanisms, such as bonding quality, local thermal history, raster deformation resistance, and defect formation, which are not fully accounted for by RD alone. The positive Pearson coefficients represent a positive relationship between the RD and the mechanical responses, indicating that as the RD increases, E, σ_peak_, and SEA tend to increase.

The findings demonstrate that the mechanical performance of FDM-fabricated lattice structures is governed not only by lattice geometry, material composition, and RD, but also by the FDM printing parameters. In particular, extrusion temperature and layer thickness significantly affect material deposition, interfacial bonding quality, and the as-built relative density, which in turn influence stiffness, strength, and energy absorption. Thus, extrusion temperature and layer thickness provide an effective means of controlling stiffness, strength, and energy absorption in lattice structures for lightweight load-bearing and energy-absorbing applications. The results further indicate that improving raster interfacial bonding reduces defect formation and delays crack initiation during compression, thereby enhancing the deformation stability and energy absorption capability of the lattice structures. In this regard, ET can be used as an effective process parameter for improving raster interfacial bonding and consequently enhancing the energy absorption capability of lattice structures. The results also demonstrate that the mechanical performance of FDM-fabricated lattice structures is highly sensitive to FDM-induced variations in as-built RD arising from the printing parameters, even when the designed RD is held constant. In this regard, it should be noted that deviations of the as-built RD from the designed value may arise not only from the lattice design, designed RD, and material composition, but also from the FDM printing parameters. Thus, the findings of this work and [32,33] demonstrate the significance of examining how several factors, such as the design, material, RD, and printing parameters, affect the printability and functionality of the lattice structures.

## 5. Conclusions

This study statistically and physically evaluated the influence of key FDM printing parameters, including ET, PS, and LT, on the mechanical behavior of D-TPMS composite structures under compression loading. Based on the results of this study, the following conclusions are drawn:

- The as-built samples closely matched the design RD (44%), with absolute errors ranging from 0.3% to 4.5%. The largest deviation (~4.5% below the design value) occurred for a low ET (205 °C) combined with a high LT (0.3 mm), likely due to insufficient melt flow and reduced contact area between adjacent rasters, which promoted gap/void formation. This highlights the considerable influence of ET and LT on lattice printability.
- Increasing ET shifts the failure mechanism from early wall and layer fracturing at a low ET to predominantly wall yielding at a high ET, reflecting improved material fusion and interfacial bonding. This delays crack initiation, promotes more stable deformation, and enhances structural integrity and energy absorption throughout compression.
- Increasing LT reduces the mechanical properties (E, σ_peak_, and SEA) and promotes gradual layer separation rather than concentrated fracturing.
- LT was the most influential parameter, accounting for 77.45% of the total variation in E (76.19% from LT and 1.26% from LT^2^) and 89.25% of the total variation in σ_peak_ (89.02% from LT and 0.23% from LT^2^). These results highlight LT as a critical FDM parameter governing the stiffness and peak strength of composite TPMS lattice structures.
- ET showed a pronounced impact on SEA by improving raster interfacial bonding and facilitating melt flow, which promoted better filling of voids and pores. Overall, ET contributed 55.76% to the total variation in SEA (48.92% from ET and 6.84% from ET^2^), indicating that ET is an effective FDM parameter for enhancing the energy absorption capability of lattice structures.
- PS showed a relatively lower impact on E and SEA, contributing 3.08% and 11.37% (11.25% from PS and 0.12% from A^2^) to the total variation, respectively, while its effect on σ_peak_ was marginal.
- Using the desirability approach, the optimal settings: 60 mm/s PS, 232 °C ET, and 0.2 mm LT, simultaneously maximized E (0.567 GPa), σ_peak_ (15.937 MPa), and SEA (15.510 J/g). Experimental results closely agreed with predictions, with a maximum percentage error of ±1.41% across all responses, validating the statistical prediction models.
- ET and LT highly influenced the as-built RD, which in turn governed the mechanical behavior of the lattice structures. This was demonstrated by correlation analysis, which revealed significant relationships between RD and the compression responses E, σ_peak_, and SEA, with correlation coefficients exceeding 0.8.
- This study enhances the understanding of how FDM printing parameters affect the mechanical properties of PLA/CFs D-TPMS lattice structures and demonstrates the ability to predict their mechanical behavior.
- The findings provide practical guidance for selecting FDM printing parameters to improve the mechanical performance of additively manufactured composite TPMS lattice structures.

Although the present study provides useful insights, it is limited to a specific TPMS geometry and material composition, and selected FDM printing parameters. Therefore, future work should extend the analysis to other lattice geometries, materials, and processing conditions to further clarify the structure–processing–property relationships in FDM-printed lattice structures. In addition, given the high correlation between the mechanical performance and FDM-induced variations in as-built RD arising from the printing parameters, future work should aim to distinguish the RD-mediated effects of the FDM printing parameters from their direct, RD-independent effects.

## Figures and Tables

**Table 1:** PLA/CFs material characteristics.

Characteristic	Value
Density	1.29 g/cm^3^
Tensile modulus	4950 MPa
Tensile strength at break	48 MPa
Tensile elongation at break	2%
Flexural strength	89 MPa
Flexural modulus	6320 MPa
Deflection temperature at 0.45 MPa	91 °C
Surface resistance	>10^9^ Ohm/sq

**Table 2:** FDM parameters and their settings.

Parameter	Levels		
	−1	0	1
Printing speed (PS), mm/s	30	45	60
Extrusion temperature (ET), °C	205	220	235
Layer thickness (LT), mm	0.2	0.25	0.3

**Table 3:** Fixed FDM process parameters used throughout all experiments.

Parameter	Value
Bed temperature	65 °C
Raster width	0.45 mm
Raster angle	±45°
Infill	100%
Number of perimeters	2
Fan speed for the 1st layer	0%
Fan speed after 3rd layer	100%

**Table 4:** RSM experiments along with the results of E, σ_peak_, and SEA.

	FDM Parameters			Mechanical Responses		
Run#	PS (mm/s)	ET (°C)	LT (mm)	E (GPa ± SD)	σ_peak_ (MPa ± SD)	SEA (J/g ± SD)
1	30	205	0.2	0.522 ± 0.004	15.032 ± 0.068	14.65 ± 0.181
2	60	205	0.2	0.535 ± 0.005	15.139 ± 0.228	14.846 ± 0.149
3	30	235	0.2	0.577 ± 0.002	15.816 ± 0.184	15.156 ± 0.202
4	60	235	0.2	0.577 ± 0.004	15.73 ± 0.134	15.366 ± 0.3
5	30	205	0.3	0.432 ± 0.002	13.373 ± 0.027	14.351 ± 0.114
6	60	205	0.3	0.464 ± 0.008	13.602 ± 0.194	14.556 ± 0.228
7	30	235	0.3	0.468 ± 0.002	13.829 ± 0.048	14.764 ± 0.001
8	60	235	0.3	0.491 ± 0.003	14.03 ± 0.088	14.958 ± 0.051
9	30	220	0.25	0.514 ± 0.011	14.537 ± 0.029	14.778 ± 0.092
10	60	220	0.25	0.535 ± 0.008	14.691 ± 0.012	15.064 ± 0.077
11	45	205	0.25	0.506 ± 0.003	14.238 ± 0.008	14.48 ± 0.101
12	45	235	0.25	0.535 ± 0.012	14.729 ± 0.102	14.916 ± 0.031
13	45	220	0.2	0.562 ± 0.013	15.624 ± 0.016	15.074 ± 0.044
14	45	220	0.3	0.475 ± 0.011	13.801 ± 0.126	14.715 ± 0.198
15	45	220	0.25	0.527 ± 0.01	14.663 ± 0.066	14.888 ± 0.019
16	45	220	0.25	0.526 ± 0.008	14.65 ± 0.076	14.817 ± 0.146
17	45	220	0.25	0.529 ± 0.013	14.608 ± 0.043	14.953 ± 0.076
18	45	220	0.25	0.521 ± 0.004	14.662 ± 0.053	15.004 ± 0.197
19	45	220	0.25	0.53 ± 0.017	14.667 ± 0.121	14.939 ± 0.051

**Table 5:** Fitting statistics of E, σ_peak_, and SEA.

Response	R^2^ (%)	Adjusted R^2^ (%)	Predicted R^2^ (%)	Adeq. Precision
E	99.46	99.02	97.67	55.99
σ_peak_	99.84	99.69	98.50	92.30
SEA	96.02	94.49	91.95	31.98

**Table 6:** Reduced ANOVA Table of E.

Source	SS	df	MS	F-Value	*p*-Value	Cont.%
Model	0.02561	8	0.003202	228.9	<0.0001	99.46
A-PS	0.0007921	1	0.0007921	56.64	<0.0001	3.08
B-ET	0.003553	1	0.003553	254.1	<0.0001	13.80
C-LT	0.01962	1	0.01962	1403	<0.0001	76.19
AB	5.51 × 10^−5^	1	5.51 × 10^−5^	3.942	0.0752	0.21
AC	0.0002311	1	0.0002311	16.53	0.002268	0.90
BC	0.0001361	1	0.0001361	9.733	0.01088	0.53
B^2^	0.000897	1	0.000897	64.14	<0.0001	3.48
C^2^	0.000325	1	0.000325	23.24	0.0007019	1.26
Residual	0.0001399	10	1.40 × 10^−5^			0.54
Lack of Fit	8.42 × 10^−5^	6	1.40 × 10^−5^	1.007	0.5218	
Pure Error	5.57 × 10^−5^	4	1.39 × 10^−5^			
Cor Total	0.02575	18				

**Table 7:** Reduced ANOVA table of σ_peak_.

Source	Sum of Squares	df	Mean Square	F-Value	*p*-Value	Cont.%
Model	8.5020	8.0000	1.0630	715.1000	<0.0001	99.84
A-PS	0.0367	1.0000	0.0367	24.6700	0.0006	0.43
B-ET	0.7560	1.0000	0.7560	508.8000	<0.0001	8.88
C-LT	7.5810	1.0000	7.5810	5101.0000	<0.0001	89.02
AB	0.0062	1.0000	0.0062	4.1780	0.0682	0.07
AC	0.0208	1.0000	0.0208	13.9900	0.0038	0.24
BC	0.0302	1.0000	0.0302	20.3100	0.0011	0.35
B^2^	0.0514	1.0000	0.0514	34.5600	0.0002	0.60
C^2^	0.0198	1.0000	0.0198	13.3100	0.0045	0.23
Residual	0.0149	10.0000	0.0015			0.17
Lack of Fit	0.0125	6.0000	0.0021	3.4710	0.1243	
Pure Error	0.0024	4.0000	0.0006			
Cor Total	8.5160	18.0000				

**Table 8:** Reduced ANOVA table of SEA.

Source	Sum of Squares	df	Mean Square	F-Value	*p*-Value	Cont. %
Model	1.0173	5	0.20346	62.71	<0.0001	96.02
A-PS	0.11915	1	0.11915	36.722	<0.0001	11.25
B-ET	0.51833	1	0.51833	159.76	<0.0001	48.92
C-LT	0.30613	1	0.30613	94.354	<0.0001	28.89
A^2^	0.0012566	1	0.0012566	0.38731	0.54448	0.12
B^2^	0.072451	1	0.072451	22.33	0.00039657	6.84
Residual	0.042179	13	0.0032445			3.98
Lack of Fit	0.02195	9	0.0024389	0.48225	0.83291	
Pure Error	0.020229	4	0.0050573			
Cor Total	1.0595	18				

**Table 9:** Mathematical prediction models of E, σ_peak_, and SEA.

Response	Mathematical Model
E (GPa) =	−1.84530 + 0.001368 × PS + 0.018601 × ET + 2.03108 × LT − 0.000012 × PS × ET + 0.007167 × PS × LT − 0.0055 × ET × LT − 0.000035 × ET^2^ − 4.05915 × LT^2^
σ_peak_ (MPa) =	−20.4476 + 0.014284 × PS + 0.337458 × ET −18.2905 × LT −0.000123807 × PS × ET + 0.0679608 × PS × LT −0.081896 × ET × LT −0.000666095 × ET^2^ + 31.6722 × LT^2^
SEA (J/g) =	−19.84633 − 0.021227 × PS + 0.311494 × ET − 3.49934 × LT + 0.000317 × PS^2^ − 0.00067344 × ET^2^

**Table 10:** Optimal FDM settings along with the experimental validation.

Parameters				Responses		
	PS (mm/s)	ET (°C)	LT (mm)	E (GPa)	σ_peak_ (MPa)	SEA (J/g)
Optimal/Prediction	60	232	0.2	0.575	15.749	15.339
Validation/Exp.	60	232	0.2	0.567 ± 0.008	15.937 ± 0.024	15.510 ± 0.074
Percentage error (%)				−1.41	1.18	1.10

**Table 11:** Pearson correlation matrix of E, σ_peak_, and SEA and RD.

		E	σ_peak_	SEA
RD	Correlation	0.839	0.854	0.818
	*p*-value	0.000	0.000	0.000

## Data Availability

The original contributions presented in this study are included in the article. Further inquiries can be directed to the corresponding author.
